# Biological Characteristics and Research Progress of the Rice Leaf Beetle *Oulema oryzae* (Coleoptera: Chrysomelidae: Criocerinae)

**DOI:** 10.3390/insects17101050

**Published:** 2026-10-12

**Authors:** Xiangqin Chen, Qiuming Chen, Shuxiang Zhou, Xinyu Xu, Junjie Yan, Ziyao Liu, Yongming Ruan, Pingyang Zhu

**Affiliations:** 1 College of Life Sciences, Zhejiang Normal University, Jinhua 321004, China; chenxiangqin11@163.com (X.C.); 19548991525@163.com (X.X.); 15669571936@163.com (J.Y.); liuziyao210@163.com (Z.L.); ruanym@zjnu.cn (Y.R.); 2 Harbin Municipal Academy of Agricultural Sciences, Harbin 150029, China; 13766833797@163.com; 3 Institute of Plant Protection, Jilin Academy of Agricultural Sciences, Gongzhuling 136100, China; df-200@yeah.net

**Keywords:** coleopteran pest, morphology traits, developmental dynamics, environmental drivers, research progress, integrated pest management

## Abstract

The rice leaf beetle, *Oulema oryzae*, is a pest across eastern Asia and eastern Russia; both its larval and adult stages cause considerable yield losses through foliar consumption. Its obligate diapause ensures population persistence through adverse seasons, but this strategy also hampers outbreak predictability and compromises timely interventions. This paper combines a narrative review with original observations. We first presented original observations on the developmental biology of *O. oryzae*. The morphological traits of all developmental instars, instar-specific diagnostic features, molting behaviors, and the complete prepupal-to-pupal transition, were described. These observations provide key criteria for accurate field identification and optimal timing of control measures. We then synthesized current knowledge on the biotic and abiotic drivers that shape population dynamics and phenology, as well as evolving integrated pest management (IPM) protocols integrating chemical, biological, and cultural tactics. Finally, we identified critical research frontiers, including the molecular regulation of diapause and development, genomic responses to climate change and cropping intensification, and multitrophic interactions with natural enemies and host plants. By integrating original observations with a broader synthetic review, this paper provides a conceptual foundation for sustainable management and actionable insights for refining IPM strategies against this economically damaging rice pest.

## Simple Summary

*Oulema oryzae* a coleopteran pest that damages rice crops across eastern Asia and Russia. This insect must go through a resting period each year to survive the cold season. In this review, we describe the beetle’s biological characteristics at each life stage, how it sheds its old skin as it grows, and how it changes from a larva into a pupa. We also bring together recent discoveries about how weather, biotic factors, and farming practices affect this beetle, along with current control methods. Finally, we suggest that future research should focus on the beetle’s internal genetic processes and how it adapts to changing environments.

## 1. Introduction

The leaf beetle subfamily Criocerinae (Coleoptera: Chrysomeloidea) encompasses approximately 1500 described species across 21 genera, including *Oulema* [1]. Over 125 species *Oulema* insects have been recorded all of the word, and 12 species occuring in China [2], including the economically damaging pests *Oulema oryzae* (Kuwayama, 1931) [3] *Oulema melanopus* (Linnaeus, 1758) [4] and *Oulema tristis* (Herbst, 1786) [5]. High morphological overlap among *Oulema* species frequently confounds traditional identification, necessitating male genitalia dissections or molecular diagnostic tools like DNA barcoding for precise species determination; however, the standard COI barcode marker cannot reliably differentiate all species within the *O. melanopus* complex [6,7].

Although the genus *Oulema* insects spans the Palaearctic, Oriental, and Afrotropical realms [8,9], *O. oryzae* is predominantly restricted to Asia, occurring across China, Japan, the Korean Peninsula, and eastern Russia [10,11]. Within China, population densities vary substantially by region. Severe infestations frequently plague northern provinces of China, such as Heilongjiang, Liaoning, Jilin, and eastern Inner Mongolia [12], whereas central provinces like Shaanxi and Hunan experience localized outbreaks [13]. Conversely, southern regions, including Taiwan, Yunnan, Guizhou, Sichuan, Fujian, Guangxi, and Xinjiang, reported only mild, sporadic occurrences [14].

Although a primary insect pest of cultivated rice (*Oryza sativa*), *O. oryzae* exhibits a broader host range within Poaceae plants, successfully feeding on wild rice (*Zizania latifolia*) and cutgrass (*Leersia sayanuka*) [15]. Both adults and larvae damage rice, though larval feeding is particularly destructive. Adults scrape mesophyll tissue between the leaf veins, generating distinctive white longitudinal streaks that cause leaves to split under. Larvae consume the mesophyll and upper epidermis while sparing the lower cuticle, creating irregular translucent patches that lead to leaf curling, desiccation, and chlorosis. Heavily infested paddies [16], resulted in stunted plant growth, reduced tillering, and typical yield losses of 5% to 20% [17].

In Heilongjiang Province, China, the rice leaf beetle completes a single univoltine generation per year governed by obligate adult diapause, with total adult longevity reaching nearly 310 days [18]. In Japan, Overwintering adults shelter in rhizosphere soil surrounding gramineous weeds on hillside slopes adjacent to paddies [19]. In Anhui Province, China, in late April, overwintered adults emerge from overwintering sites in non-crop weed habitats, not from paddies, as ambient temperatures stabilize above 15 °C in late April, and then migrate into paddies by mid-May to feed, mate, and oviposit. Reproductive activity peaks at approximately 21 °C. Larvae hatch in paddies in mid-May, pupation occurs in paddies from late May through early June, and a new generation of adults emerges from early July onward. By September, these adults return to non-crop weed habitats to undergo winter diapause [20].

To date, research on *O. oryzae* has focused mainly on field biology, phenotypic observations, and empirical control [21], however, its molecular physiology, genetic adaptation, and ecological interactions remain poorly understood. Although it is currently a major pest in East Asia, *O. oryzae* may threaten rice-growing regions beyond its current range as global warming reshapes rice production. Given this potential risk, this narrative review summarizes its biological traits and recent progress, and identifies future directions for sustainable management.

## 2. Morphological Characteristics of *O. oryzae*

*Oulema oryzae* adults were collected from paddies in Shangzhi City, Heilongjiang Province, China (128°1.77′ E, 45°15.40′ N). In the laboratory, colonies were maintained on 30-day-old ‘Daohuaxiang 2’ rice seedlings within a climate chamber maintained at 24 ± 2 °C, 70 ± 5% relative humidity, and a 14:10 h (L:D) photoperiod. Unless otherwise stated, all morphological data, measurements, images, and observations presented in this section are original and were obtained by the authors from laboratory-reared specimens.

### 2.1. Body Length and Width Across Developmental Stages

Morphological features and dimensions for each stage (adults and larvae) were quantified using a VHX 500FE digital microscope (KEYENCE). For each developmental stage, 20 individuals were randomly selected for repeated measurements. Adult body length (from the anterior margin of the head to the elytral apex) was measured, whereas larval length was recorded as the straight-line distance from the anterior head margin to the abdominal tip. Body width represents the maximum transverse distance for all stages. Raw measurements and photographs of adults with a scale bar are provided in Supplementary Table S1.

Female adults were slightly larger than males, measuring 4.42 ± 0.26 mm in length and 1.58 ± 0.06 mm in width; males measured 3.97 ± 0.25 mm in length and 1.43 ± 0.07 mm in width. Elliptical eggs measured 0.71 ± 0.05 mm in length and 0.34 ± 0.02 mm in width. Larval body length and width progressed through four instars: 1st instar, 0.87 ± 0.12 mm × 0.37 ± 0.03 mm; 2nd instar, 1.45 ± 0.17 mm × 0.70 ± 0.06 mm; 3rd instar, 2.07 ± 0.20 mm × 0.94 ± 0.21 mm; and 4th instar, 3.82 ± 0.32 mm × 1.48 ± 0.08 mm. Constructed cocoons measured 4.83 ± 0.39 mm in length and 2.30 ± 0.17 mm in width. All values are presented as mean ± SD based on the authors’ own measurements *(n* = 20 per group unless otherwise stated).

### 2.2. Stage-Specific Morphological Traits and Instar Development

**Figure 1:**
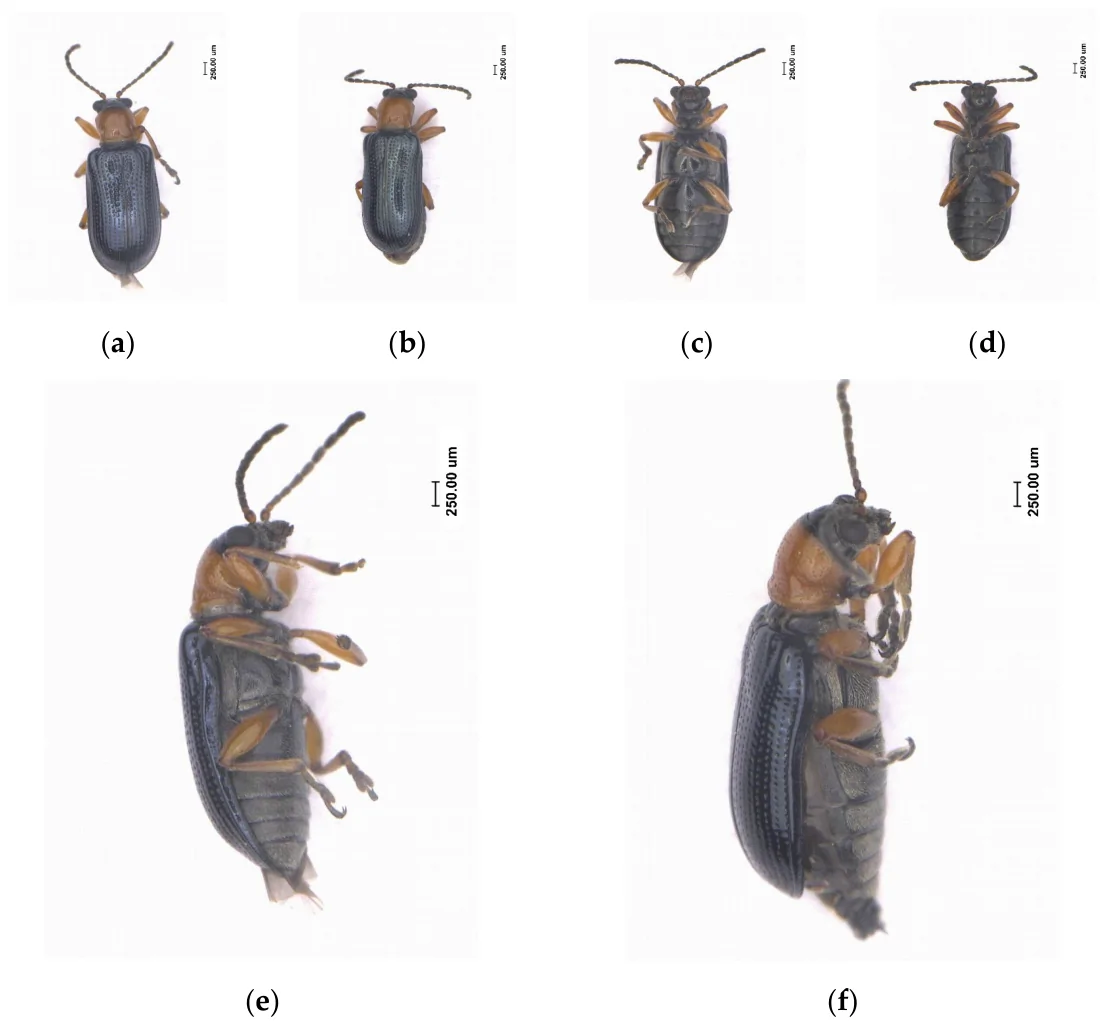
Morphology of an adult rice leaf beetle. Dorsal views of male (**a**) and female (**b**); ventral views of male (**c**) and female (**d**); lateral views of male (**e**) and female (**f**).

Adults: Adults feature a black head with chewing mouthparts and dark brown, moniliform antennae composed of a scape, pedicel, and a nine-segmented flagellum (Figure 1a). The sub-bell-shaped pronotum is yellowish-brown (Figure 1b). The legs are predominantly yellowish-brown with dark brown coxae, trochanters, tarsi, and claws (Figure 1c). The abdomen tapers posteriorly across five visible sternites; the first sternite is widest, sternites 2–4 narrow progressively, and the fifth broadens slightly to support the terminal anal segment (Figure 1d). The metallic, bluish-black elytra each carry 10–11 longitudinal rows of punctures, concealing transparent, membranous hindwings with distinct venation capable of short-distance flight [19] (Figure 1e,f).

**Figure 2:**
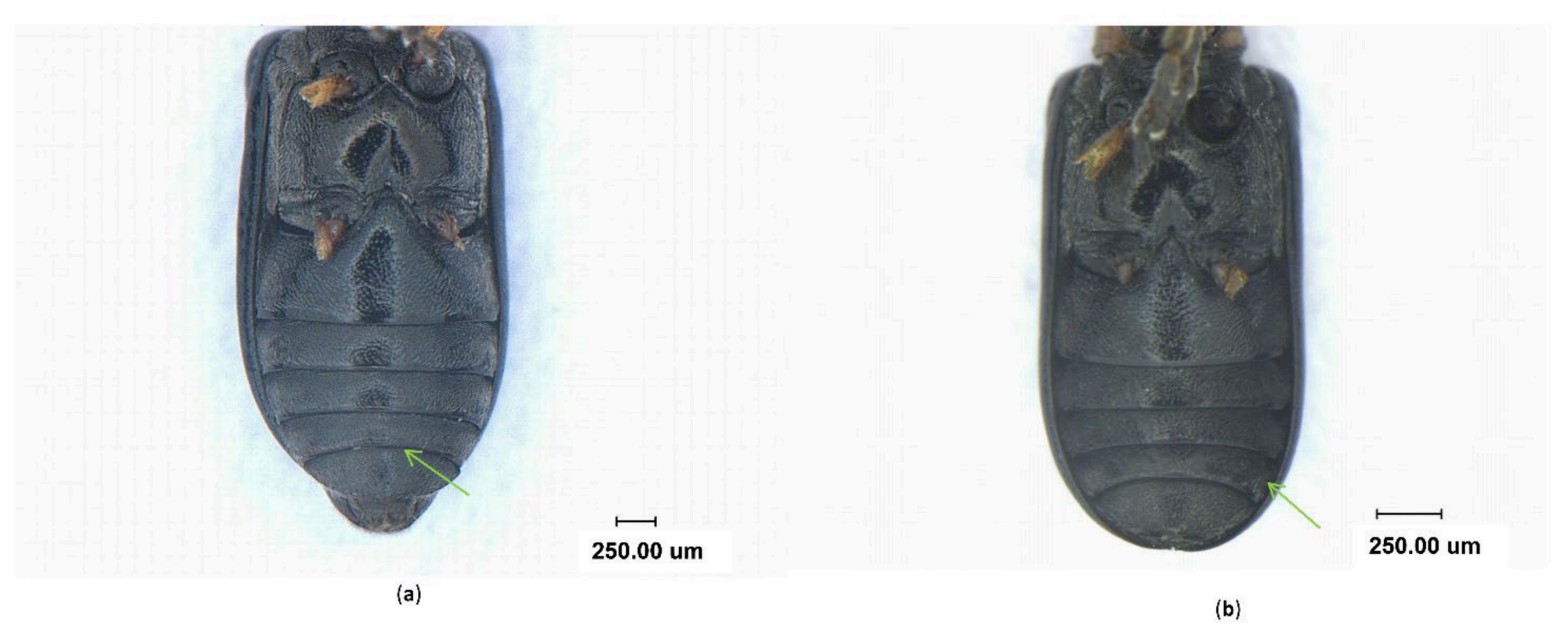
Ventral view of female (**a**) and male adult (**b**), Green arrows indicate the boundary between ventrites 4 and 5.

During the breeding season, females are readily identified by their exposed ovipositors. Outside the reproductive period, sexual dimorphism relies on abdominal structure and overall body shape: females are larger with distended abdomens, whereas males are slender with flattened abdomens. In females, the mean ratio of the midline length of ventrite 4 to that of ventrite 5 was 0.64 *(*Table S2, Figure S2a), and the 6th ventrite projects slightly to give the posterior abdomen a truncated lateral profile (Figure 2a). In males, this ventrite 4/ventrite 5 ratio was 0.54 (Table S2, Figure S2b), resulting in a straighter abdominal apex with minimal curvature (Figure 2b).

**Figure 3:**
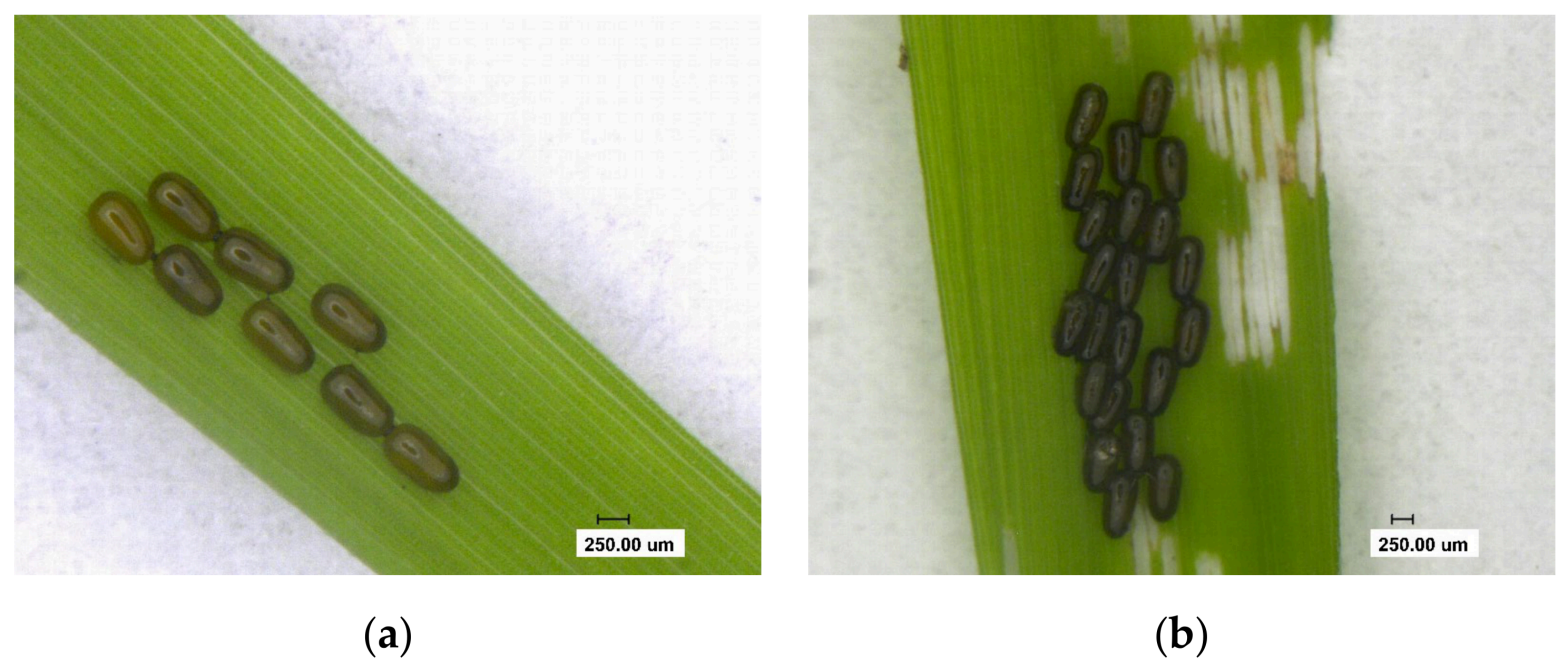
Freshly laid eggs (**a**) and nearly hatched eggs (**b**).

Eggs: Females typically deposit cluster-laid, elliptical eggs (~20 per clutch) on leaf blades. Freshly laid eggs are bright yellow, turning dark brown within minutes (Figure 3a) and shifting to yellowish-brown shortly before hatching (Figure 3b). Egg incubation lasts 4–8 days under field conditions (typically 6 days).

**Figure 4:**
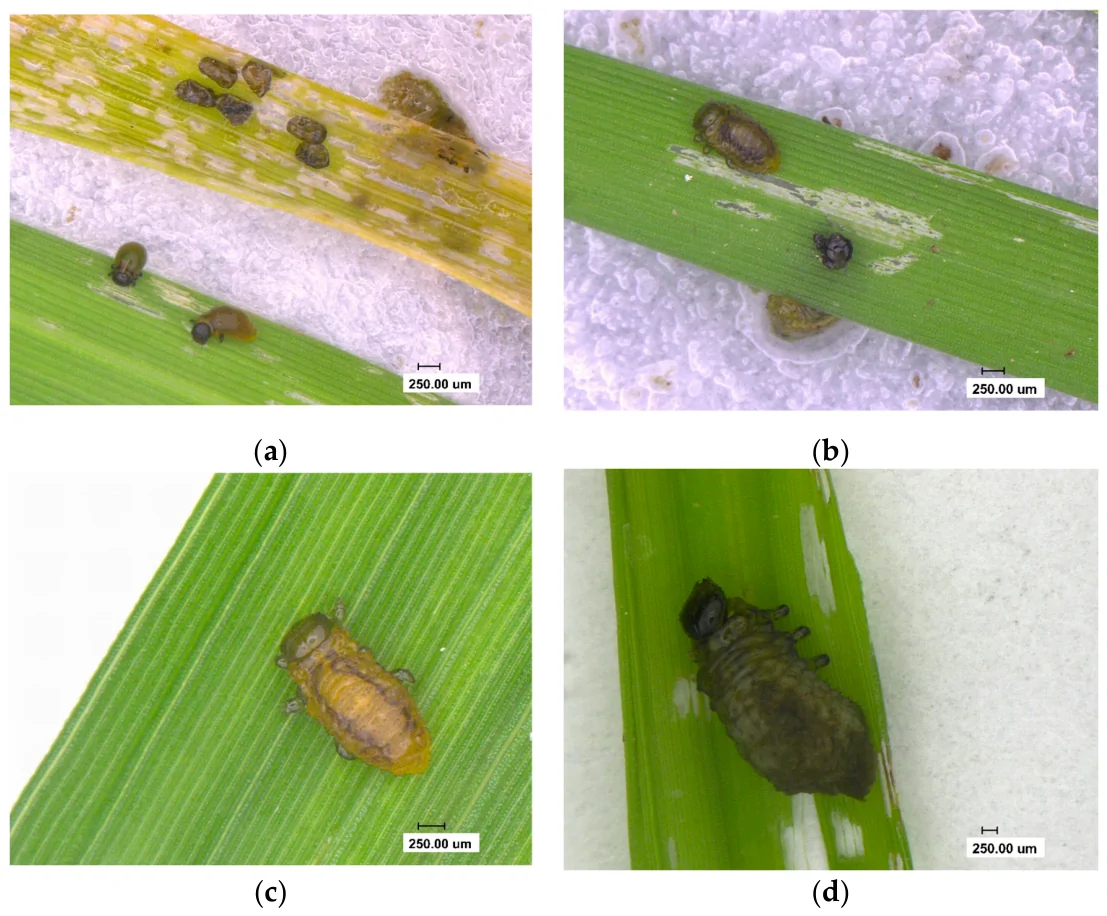
Different stages of *Oulema oryzae* larvae, newly hatched larvae (**a**), second instar larva (**b**), third instar larva (**c**), and fourth instar larva (**d**).

Larvae are generally pale yellow, the black, finely pubescent head houses chewing mouthparts that scrape mesophyll tissues. (Figure S2a). The dorsal abdomen is strongly convex, peaking in girth at the third segment before tapering posteriorly to form a semi-gourd shape (Figure S2b). The dorsal anus excretes grayish-yellow to dark green fecal matter that accumulates over the dorsum (Figure S2c). Hydrated by leaf dew, this fecal shield swells, imparting a droplet-like appearance that deters predators and prevents desiccation Figure S2d). First instar: Newly hatched larvae possess a dark brown head and pale-yellow body. Following a brief post-eclosion rest, neonates disperse to begin scraping leaf surfaces (Figure 4a). Second instar: Second-instar larvae are more mobile, actively moving between leaves to expand feeding areas (Figure 4b). Third instar: Third-instar larvae feed voraciously, rapidly consuming large areas of leaf mesophyll (Figure 4c). Fourth instar: Semi-pear-shaped fourth instars display dark black head capsules, thoracic legs, and abdominal markings against a yellowish-brown body. In our observations, fourth instars were the most destructive stage, feed heavily on mesophyll tissues and presumably accumulating energy for cocooning and pupation (Figure 4d). Larval nutrition may have carry-over effects on adult reproduction. Previous studies have shown that Lepidoptera larval feeding also supports ovarian enlargement [22].

**Figure 5:**
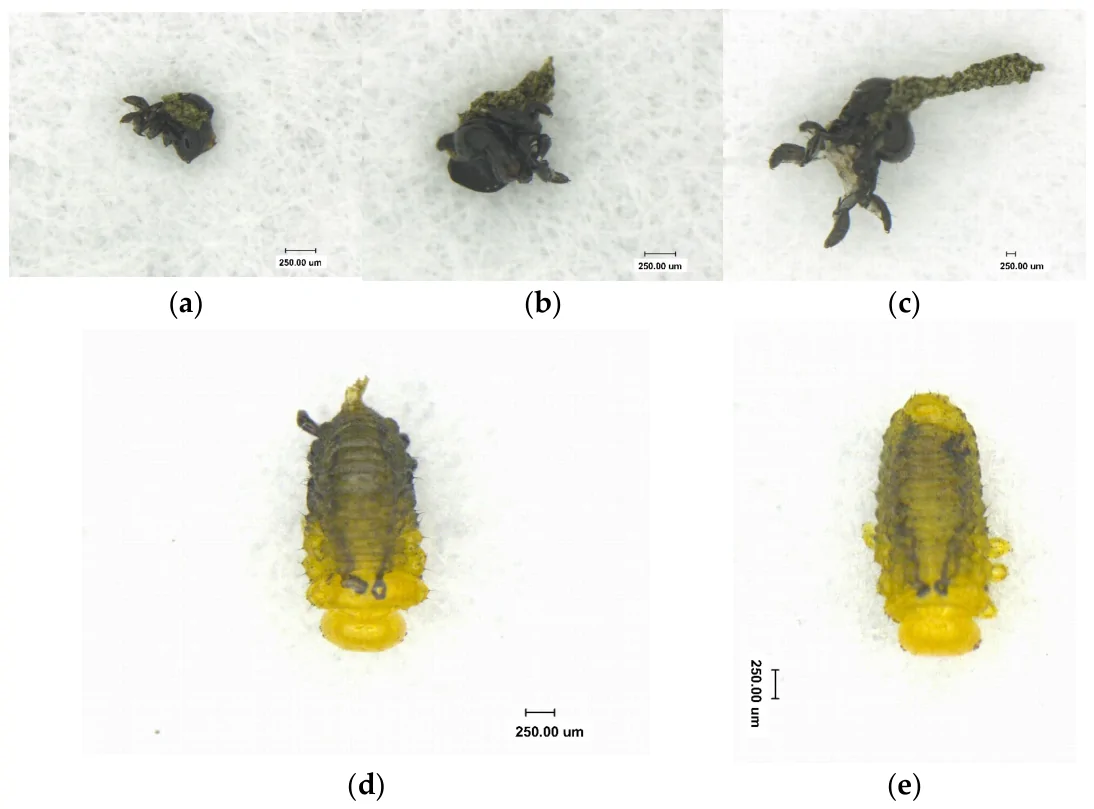
The first (**a**), second (**b**), third (**c**) larval molts. A molting larva (**d**) and a molted larva (**e**).

Larval molting: *O. oryzae* has four larval instars and undergoes three larval molts (Figure 5a–c). Before each molt, larvae cease feeding andanchor firmly to the leaf surface, with the ventral side flattened and the dorsum raised. The old cuticle then splits along the mid-dorsal thorax, the larva elevatesits bod pulls its head and thorax out of the old cuticle; during this process, the accumulated dorsal fecal matter is shed together with the old cuticle. The larva then pulls its legs free and crawls forward to complete ecdysis (Figure 5d). Newly molted larvae initially exhibit golden-yellow heads and prolegs with orange-yellow bodies (Figure 5e). The head capsule and legs subsequently sclerotize into a dark brown shade matching mature instars of that stage.

**Figure 6:**
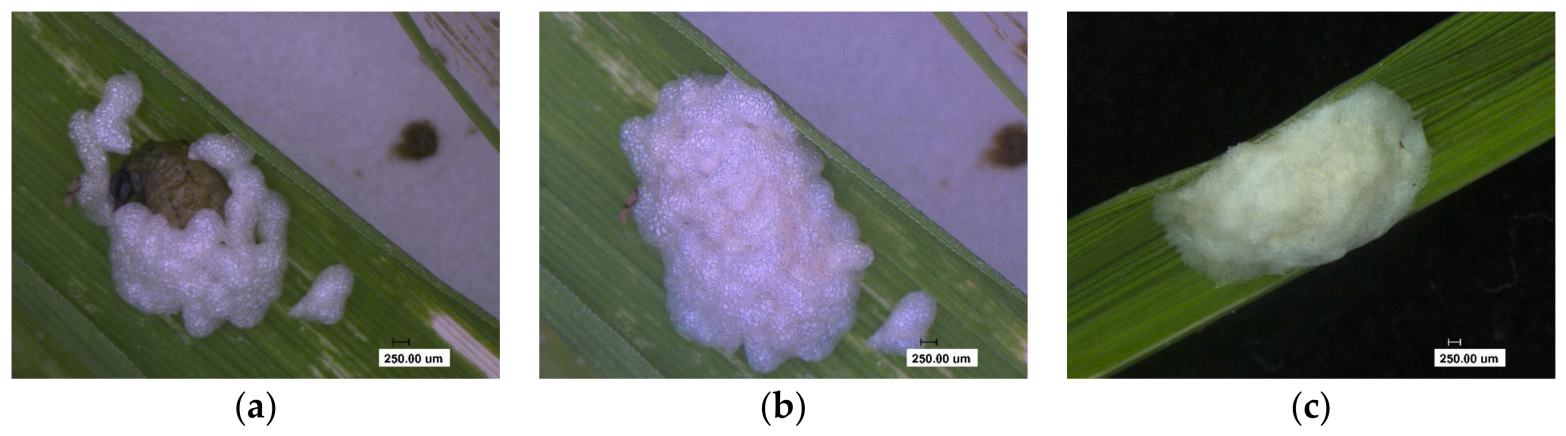
Larva spinning a cocoon (**a**), a freshly spun cocoon (**b**), and a 4-day-old cocoon (**c**).

Cocoon: In our laboratory observations, larvae constructed cocoons by spinning oral foam secretions around themselves; this process was observed to be completed within approximately 3–4 min (Figure 6a). The resulting grayish-white, cotton-like structures measured 4.3–5.6 mm in length and 1.8–2.5 mm in width (Figure 6b,c).

**Figure 7:**
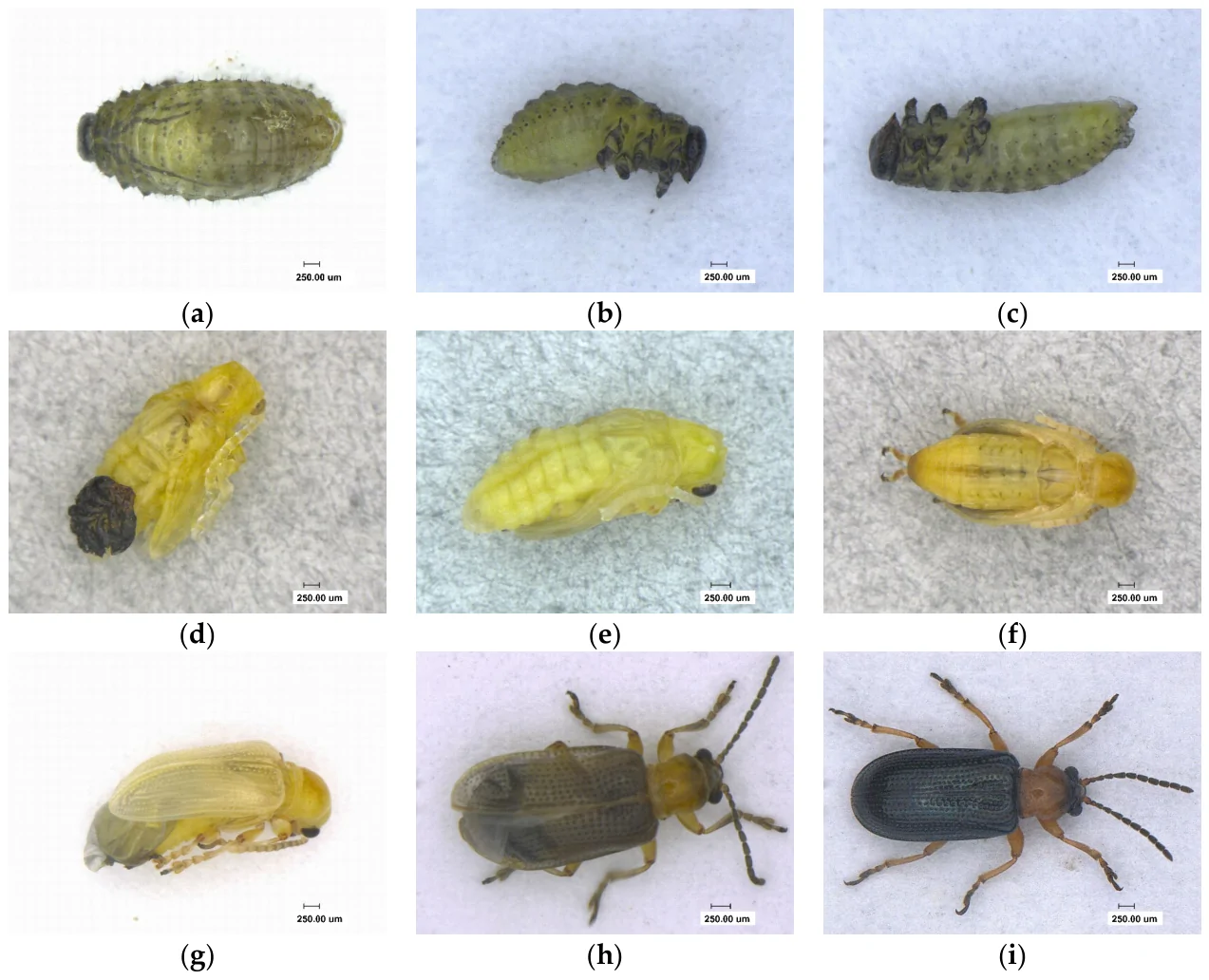
Prepupal ((**a**–**c**), days 1–3), late pupal ((**d**–**h**), days 4–8), and newly emerged adult ((**i**), day 9) stages of *Oulema oryzae*.

Pupa: The pupal phase lasts 5–11 days (typically 8 days), during which radical metamorphosis occurs. Inside the new cocoon, the insect initially remains in a larval-like state termed the prepupa [23,24]. Prepupa: On Day 1, prepupae remain active; when removed from their cocoon, they can construct a new cocoon (Figure 7a). By Day 2, prepupae become soft and immobile, when if removed from their cocoon, unable to survive. (Figure 7b). By Day 3, prepupae stiffen, exhibiting distinct body segmentation; a fraction of individuals removed at this stage can successfully complete adult development (Figure 7c). Exarate pupa: Shedding the final larval cuticle reveals a golden-yellow exarate pupa. Pupal metamorphosis progresses across distinct developmental phases before emergence: Initial pupal stage: Moniliform antennae appear translucent, tarsal segmentation and elytra are incomplete, compound eyes turn light brown, and mouthpart structures remain undifferentiated (Figure 7d and Figure S3a). Early pupal stage: Compound eyes darken to deep brown. Leg segments become distinct with brick-red articulations, while tarsi and mandibles turn dark brown (Figure 7e and Figure S3b). Middle pupal stage: Overall pigmentation deepens. Antennae display 11 distinct segments, legs show fully formed femora, tibiae, and tarsi, forewings remain unsclerotized, hindwings remain compact, and mouthparts complete differentiation (Figure 7f and Figure S3c). Late pupal stage: The entire pupal body turns golden-yellow. Soft forewings and fully expanded, longer hindwings extend to cover the abdomen, completing pre-emergence development (Figure 7g and Figure S3d). Early emergence stage: Near the end of pupation, antennae display 11 fully extended, dark brown segments. The legs turn yellowish-brown with black tarsi, and the dark elytra develop transparent, punctate patterns. The head turns black while the thorax and abdomen remain yellowish (Figure 7h and Figure S3e). Emergence: Upon completing development, adult *Oulema oryzae* chew through the cocoon wall with their mandibles, then emergence (Figure 7i and Figure S3f).

## 3. Research Progress on *O. oryzae*

### 3.1. Impacts of Abiotic and Biotic Factors

#### 3.1.1. Abiotic Drivers

Temperature and humidity primarily dictate the population dynamics of *O. oryzae* [25]. Thermal performance models established minimum developmental thresholds for eggs, larvae, and pupae at 8.39 °C, 7.20 °C, and 7.23 °C, respectively, with corresponding thermal constants of 53.01, 118.31, and 122.36 degree-days [26]. Field monitoring demonstrated that seasonal occurrence hinges on the mean temperature, photoperiod, and rainfall patterns, with weather conditions between June 21 and 30 exerting a pivotal influence, as cool early-season temperatures followed by late-June warming strongly promote population growth in Tonghua City, Jilin Province, China [26].

#### 3.1.2. Biotic Drivers

Biotic interactions critically shape population fluctuations and behavioral adaptation. Deciphering how *O. oryzae* interacts with rice hosts offers essential insights for resistance breeding. Comparative studies on the resistant cultivar ‘Suijing 4’ and susceptible ‘Daohuaxiang 2’ revealed that larval feeding triggers antioxidant enzyme activities involving superoxide dismutase (SOD), peroxidase (POD), and catalase (CAT) and alters malondialdehyde (MDA) levels. Under herbivore pressure, SOD activity rises continuously, whereas POD, CAT, and MDA levels peak before declining [27]. Feeding stress concurrently depletes total protein and soluble sugars while elevating free proline concentrations, for example, after 24 h of infestation, the soluble sugar content of susceptible plants dropped from 5.35 to 4.09 mg/g, and that of resistant varieties from 5.58 to 4.75 mg/g [28]. Furthermore, volatile and non-volatile secondary metabolites from resistant plants exert stronger repellent and antifeedant effects against both larvae and adults than those from susceptible plants, for example, the repellency rates of healthy plants of ‘Suijing 4’ and ‘Daohuaxiang 2’ were 43.7% and 33.0%, and the antifeedant rates at 1000 mg/L of non-volatile secondary metabolites were 60.39% and 48.25%, respectively [29]. Exudate components such as adenine, adenosine, and guanine actively modulate beetle feeding behavior, with specific concentrations of adenine and its nucleotides acting as strong feeding stimulants.

At the molecular level, in contrast to the *O. oryzae*-rice system, plant–beetle interactions in other plant-herbivore systems involve tightly regulated signal transduction and metabolic reprogramming [30]. For example, Colorado potato beetles use saliva to modify host plant defense mechanisms [31]. In several crop-herbivore systems, resistant cultivars maintain robust defenses by modulating jasmonic acid signaling pathways and upregulating downstream defense genes, including terpenoid synthases [32]; However, these pathways remain completely uncharacterized in *O. oryzae*; whether they operate against *O. oryzae* is unknown.

Direct evidence for salivary effectors in *O. oryzae* is currently lacking. By analogy with chewing lepidopteran larvae, in which salivary effectors can either suppress or elicit host defenses [33], However, no salivary effectors have been identified in *O. oryzae* to date, and whether *O. oryzae* uses such effectors to modulate host defenses remains unknown. The host targets of *O. oryzae* salivary proteins also remain uncharacterized, limiting our understanding of the molecular basis of host–insect. In addition, symbiotic microbes can mediate leaf beetle fitness, as shown in the congener *O. melanopus*, that endosymbionts of *O. melanopus* enhanced host plant utilization by modulating digestive enzymes [34], degrading toxic secondary metabolites [35], or suppressing plant defense signals [36]. In contrast, the functional contributions of the microbiome to *O. oryzae*-host interactions remain unexplored. Whether *O. oryzae* harbors functionally analogous symbionts are unknown and should be tested experimentally.

#### 3.1.3. Multifactorial Ecological Interactions

In herbivore systems other than the *O. oryzae*-rice systems, abiotic and biotic pressures act synergistically through complex ecological cascades to constrain herbivore fitness and niche dynamics. Across crop–herbivore systems, soil nutrient status modifies host plant physiology and indirectly alters interactions between herbivores and higher trophic levels [37]. For instance, in rice systems, heavy nitrogen fertilization significantly accelerates the population growth of the aphid *Hysteroneura setariae* [38], whereas organic fertilization under equivalent nutrient conditions suppresses the white-backed planthopper *Sogatella furcifera* [39]. In contrast to sap-sucking herbivores, leaf-chewing insects herbivores-a group that includes *O. oryzae*-are strongly influenced by plant nitrogen content [40]. These opposing outcomes, observed in non-*Oulema* herbivores, indicate that fertilizer rate and source reconfigure host palatability and herbivore performance.

Climatic variables like temperature may similarly alter chemical signaling among plants, herbivores, and natural enemies. For example, thermal stress alters volatile organic compound (VOC) emission profiles in potato plants, disrupting natural enemy searching efficiency and weakening top-down biological control [41].

Together, these findings suggest that thermal stress may modulate plant-mediated interactions and biological control Direct multi-factor interaction studies in *O. oryzae* are currently lacking. Therefore, these findings from other herbivore systems should be treated as a hypothesis-generating framework rather than as direct evidence for *O. oryzae*; future studies should test whether similar abiotic factors shape *O. oryzae* performance and its interactions with natural enemies.

### 3.2. Pest Management Advances for O. oryzae

#### 3.2.1. Economic Thresholds

Establishing economic action thresholds is essential for *O. oryzae* management, as pest density drives damage and yield loss. However, thresholds are context-dependent rather than universal. Sun [42] and Kong [43] found strong density–damage–yield loss relationships in Heilongjiang and Anhui, China, but these relationships were established under different regional climates, cultivars, planting dates, and pest generations. In a two-year seedling-stage study conducted in Heilongjiang Province, China, Xu [17] modeled yield loss (%) = 0.5793 + 0.0333 × larval density per 100 hills; this model requires validation before use at other crop stages or in other systems. Koyama [44] showed that larval damage reduced yield mainly by suppressing panicle formation per hill, and reported action thresholds of 0.5 egg masses per hill in early June or >20% leaf damage by late June. These thresholds may be transferable only to similar early-season rice systems, and local validation is needed elsewhere. Therefore, threshold differences arise from biological, agronomic, environmental, and economic variation, and their applicability should be defined explicitly.

#### 3.2.2. Host Resistance and Cultural Control

Integrating host resistance with tailored agronomic practices offers a sustainable strategy to suppress beetle populations. Systematic screening by Jin [45] classified cultivar resistance to *O. oryzae*, identifying ‘Suijing 4’ as resistant, ‘Fengyuan 2’ and ‘Kongyu 131’ as moderately resistant, ‘05345’ as moderately susceptible, and ‘Kenjian 5’ alongside ‘Daohuaxiang 2’ as highly susceptible. These evaluations provide clear germplasm targets for breeding programs. Additionally, field practices strongly influence pest pressure. Zeng showed that heavy nitrogen applications significantly increase infestation severity, whereas shallow water management mitigates damage. Intermittent shallow irrigation combined with timely drainage suppresses oviposition and reduces both egg hatchability and larval survival [46].

#### 3.2.3. Chemical Resistance

Chemical insecticides have long served as the primary control tactic for *O. oryzae*, but intense selective pressure has driven severe resistance, with median lethal dose (LD50) assays showing >28,000-fold resistance to carbamates (propoxur, fenobucarb, and benfuracarb) and a >50,000-fold reduction in acetylcholin-esterase (AChE) sensitivity to carbamates in resistant beetles, largely due to target-site insensitivity [47] Beyond target-site mutations, metabolic detoxification plays a critical role. Exposure to β-cypermethrin, abamectin, or imidacloprid differentially induces carboxylesterase (CarE) and glutathione S-transferase (GST) activities in larvae, with abamectin significantly elevating CarE activity [48]. Mitigating resistance requires integrated management strategies that combine distinct insecticide modes of action with targeted application timing.

#### 3.2.4. Biological Control Strategies

Biological control relies on conserving or releasing natural enemies and applying biopesticides. Documented parasitoids targeting *O. oryzae* include the egg parasitoids *Anaphes nipponicus* and *Anaphes flavipes* [10], the larval parasitoid *Lemophagus japonicas* [49], the pupal parasitoids *Oryzae Kamijo* and *Bathythrix kuwanae* [50], and the prepupal parasitoids *Pteromalus cyclostomus* and *Spaniopus monospilus* [51]. Surveys in *O. oryzae* habitats along the southwestern slopes of Changbai Mountain, China, identified 37 potential predatory species, notably *Nabis sinoferus* and *Hippodamia tredecimpunctata*, with spiders and staphylinid beetles dominating the predator complex [52]. Despite this rich natural enemy community, practical strategies for their conservation and augmentative release remain underdeveloped.

For the congener *O. melanopus*, however, biological control is far more advanced. The key larval endoparasitoid of *O. melanopus*, *Tetrastichus julis*, preferentially attacks late instars; a 48-h host exposure period optimizes both parasitism rates and mass-rearing efficiency [53]. Field trials in *O. melanopus* systems have shown that crop uniformity stabilizes the spatial distribution of *O. melanopus* and *T. julis*, lowering yield loss risks [54]. At a landscape scale (500 m radius), increased crop diversity suppresses *O. melanopus* populations by diluting host availability while expanding habitats for natural enemies [55]. Molecular gut-content analyses of *O. melanopus* confirmed that generalist predators (carabids, coccinellids, and nabids) actively consume *O. melanopus* eggs and larvae, achieving a 24-h egg predation rate of 24.5% and gut-positivity rates up to 43% [56]. *O. oryzae* and *O. melanopus* share several key biological traits, including a graminaceous host range, larval fecal shields, univoltine life cycles, and adult overwintering. Therefore, findings from *O. melanopus* may provide a useful roadmap for *O. oryzae* biocontrol. However, this extrapolation should be regarded as a hypothesis requiring validation in *O. oryzae*.

Biopesticides represent another eco-friendly component of integrated pest management (IPM). Botanical and microbial formulations are often considered environmentally safer and less prone to resistance development than synthetic insecticides. For example, d-limonene (a primary constituent of *Citrus sinensis* essential oil) exhibits potent contact toxicity against *O. melanopus* larvae, causing 85% mortality within 48 h, with rapid degradation and low residue persistence. However, the efficacy of d-limonene against *O. oryzae* has not been tested [57]. Nevertheless, high-efficacy and species-specific biological control agents or biopesticides for *O. oryzae* remain limited. Further research into their mechanisms, field application protocols, and compatibility with natural enemies is needed before widespread adoption.

## 4. Perspectives

Research on *Oulema oryzae* has built a foundational framework covering classical morphology, seasonal occurrence, and chemical control, establishing baseline support for field monitoring. Nevertheless, the current literature remains largely confined to phenotypic descriptions and applied management. Fundamental scientific questions in *O. oryzae*, such as the molecular networks driving physiology, the genetic mechanisms underpinning population adaptation, and the ecological principles governing host-environment interactions, remain unaddressed. This knowledge gap may limit the development of precise and sustainable management protocols. Future research could help address these limitations by integrating genomics, proteomics, chemical ecology, and population genetics across several promising research avenues.

### 4.1. Deciphering Eco-Physiological Interactions and Developing Ecological Control Strategies

Population dynamics of *O. oryzae* are likely shaped by both intrinsic genetics and broader agroecosystem processes, although direct evidence remains limited. Moving beyond single-factor evaluations, future research should systematically dissect host–insect interactions mediated by environmental drivers in *O. oryzae*. Priorities include evaluating how nitrogen regimes, thermal fluctuations, and photoperiod shifts regulate both host defense expression and *O. oryzae* behavior. Coupling multi-factor trials with metabolomic profiling could help clarify the mechanisms governing induced rice resistance and *O. oryzae* behavioral responses, offering an empirical foundation for optimizing fertilizer and water regimes while leveraging microclimatic manipulation for sustainable pest suppression.

### 4.2. Unraveling the Molecular Regulation of Obligate Diapause for Predictive Modeling

The univoltine life cycle and unique overwintering strategy of *O. oryzae* have long hindered the elucidation of its population dynamics. To date, the physiological pathways and genetic circuits governing diapause induction, maintenance, and termination remain uncharacterized in *O. oryzae*. Leveraging modern omics and computational modeling, future studies should pinpoint core regulatory genes in *O. oryzae*. Combining photothermal simulations with fat body histology and respirometry could help delineate key diapause transitions in *O. oryzae*. Furthermore, transcriptomic and whole-genome profiling to help identify stage-specific candidate genes in *O. oryzae* in the future studies. Their potential roles as diapause “switches” could then be explored by dsRNA-mediated RNA interference (RNAi). Integrating these molecular drivers into diapause models may enable predictive tools based on diagnostic molecular markers, potentially surpassing traditional degree-day models to improve forecasting accuracy.

### 4.3. Decoding Chemosensory Communication and Identifying Behavioral Modulation Targets

While host resistance varies among rice cultivars, the neuromolecular mechanisms by which *O. oryzae* perceives volatile and non-volatile secondary metabolites during host selection, feeding, and oviposition remain unknown. Future work should systematically annotate the chemosensory gene families of *O. oryzae*. Combining transcriptome sequencing across chemosensory tissues (antennae, maxillary palps, and legs) with homology-based searches could catalog olfactory receptors, gustatory receptors, and odorant-binding proteins in *O. oryzae*, followed by tissue-specific expression profiling via RT-qPCR. Electrophysiological recordings (EAG/SSR) coupled with behavioral assays could then screen bioactive candidate volatiles for repellent or attractant properties in *O. oryzae*. These efforts may identify lead compounds for plant-derived semiochemical formulations and could provide gene-editing targets to breed behavior-modifying, pest-resistant rice lines.

### 4.4. Mapping Population Genetics and Environmental Adaptation via Whole-Genome Sequencing

Marked regional disparities exist in damage severity and outbreak timing of *O. oryzae* between northern and southern China. Genomic evidence is needed to determine whether these variations stem from population structure, historical invasion routes, or localized adaptation, including insecticide resistance. Future research should target two primary areas: (1) Whole-genome variation: Resequencing representative geographic populations across major Chinese rice zones could map genetic structure, gene flow, and demographic history to resolve dispersal pathways of *O. oryzae*. (2) Adaptive gene discovery: Integrating regional climate data (mean temperatures, extreme cold events, rainfall, and sunshine duration) with genomic profiles could identify loci underlying latitudinal and climatic adaptation in *O. oryzae*. Particular focus should be placed on allelic variation in stress-response genes, such as heat shock proteins (HSPs), antifreeze proteins (AFPs), and trehalose synthases, to guide tailored, region-specific management strategies for *O. oryzae*.

### 4.5. Harnessing Microbiome Function for Novel Pest Control Paradigms

Associated microbiomes may critically influence nutrient acquisition, immune defense, and stress tolerance. However, the core symbiotic microbiota of *O. oryzae* and its roles in detoxifying host defenses, conferring environmental resilience, and modulating insecticide susceptibility remain uncharacterized. Future research should prioritize four areas: (1) Microbiome profiling: Employing 16S/ITS amplicon and metagenomic sequencing across populations, developmental stages, and tissues could characterize microbial diversity and identify core taxa via network analysis in *O. oryzae*. (2) Functional validation: Generating aposymbiotic lines via antibiotic treatments, validated by fluorescence in situ hybridization (FISH), could allow direct comparisons of host fitness, stress tolerance, and insecticide sensitivity to determine symbiotic contributions in *O. oryzae*. (3) Metabolic integration: Combining metagenomic functional annotations with host transcriptomics could clarify microbial roles in nitrogen recycling, cellulose breakdown, and xenobiotic degradation in *O. oryzae.* (4) Symbiont-based control: Paratransgenesis could be explored to engineer symbionts expressing pest-suppressive effectors, such as dsRNA or crystalline toxins, in *O. oryzae*. Alternatively, targeted inhibitors could be screened to disrupt essential symbionts of *O. oryzae*, impairing host nutrition and increasing susceptibility to entomopathogens such as *Beauveria bassiana* or *Metarhizium anisopliae*.

## Data Availability

All data generated or analyzed during this study are included in this published article and its supplementary information files. Further inquiries can be directed to the corresponding author.

## Supplementary Materials

The following supporting information can be downloaded at https://www.mdpi.com/article/10.3390/insects17101050/s1. Table S1. Body length and width of *Oulema oryzae*. Table S2. Mean ratio of the midline length of ventrite 4 to that of ventrite 5 in female and male *Oulema oryzae*. Figure S1. Abdomen of the female (**a**) and male (**b**) adult; Figure S2. Mud-carrying larvae at the newly hatched (**a**), second (**b**), third (**c**), and fourth (**d**) instar stages; Figure S3. Abdomen of 4–8-day post-eclosion adults (**a**–**e**) and newly emerged adults (**f**) of the rice leaf beetle (*Oulema oryzae*).

## References

[1] Caroline S.C., Schmitt M. (2015). Beetles (Coleoptera) of Peru: A survey of the families. Chrysomelidae: Criocerinae. J. Kans. Entomol. Soc..

[2] Yang X. (2024). Systematics of Subfamily Criocerinae in China (Insecta: Coleoptera: Chrysomelidae) and Evolutionary Relationship with Their Host Plants. Ph.D. Thesis.

[3] Kuwayama S. (1931). *Lema oryzae* sp. nov. Insecta Matsumurana.

[4] Bezdek J., Baselga A. (2015). Revision of western Palaearctic species of the *Oulema melanopus* group, with description of two new species from Europe (Coleoptera: Chrysomelidae: Criocerinae). Acta Entomol. Musei Natl. Pragae.

[5] Herbst J.F.W. (1786). Erste Mantisse zum Verzeichniß der ersten Klasse meiner Insektensammlung. Arch. Der Insectengeschichte.

[6] Haouas D., Mdelle L., Barhoumi W., Torres D.M. (2024). Recent data on the biology and population dynamics of *Oulema hoffmannseggii* (Coleoptera: Chrysomelidae) and its associated parasitoids. Int. J. Pest Manag..

[7] Ihrig R.A., Herbert D.A., Van Duyn J.W., Bradley J.R. (2001). Relationship between cereal leaf beetle (Coleoptera: Chrysomelidae) egg and fourth-instar populations and impact of fourth-instar defoliation of winter wheat yields in North Carolina and Virginia. J. Econ. Entomol..

[8] Piesik D., Lemńczyk G., Skoczek A., Lamparski R., Bocianowski J., Kotwica K., Delaney K.J. (2011). *Fusarium* infection in maize: Volatile induction of infected and neighboring uninfected plants has the potential to attract a pest cereal leaf beetle, *Oulema melanopus*. J. Plant Physiol..

[9] Leroy J., Chapelin-Viscardi J.-D., Genson G., Haran J., Pierre E., Streito J.-C. (2020). Distinct barcodes for the cereal leaf beetles *Oulema melanopus* and *Oulema duftschmidi* (Coleoptera: Chrysomelidae), two syntopical sibling species. EJE.

[10] Triapitsyn S.V., Rugman-Jones P.F., Kusuhara H., Nakano R., Jansta P., Arikawa S., Adachi-Hagimori T. (2023). Genetic analysis reveals conspecificity of two nominal species of *Anaphes fairyflies* (Hymenoptera: Mymaridae), egg parasitoids of *Oulema* leaf beetle (Coleoptera: Chrysomelidae) pests of cereal crops in Europe and of rice in East Asia. PLoS ONE.

[11] Andow D.A., Hidaka K. (1998). Yield loss in conventional and natural rice farming systems. Agric. Ecosyst. Environ..

[12] Jiang W., Sui W.Z. (2017). Identification and control of major rice pests. Agric. Jilin.

[13] Liu X.G. (2013). Identification and control of rice leaf beetle in Hanzhong. Plant Health Med..

[14] Samková A., Hadrava J., Skuhrovec J., Jansta P. (2020). Host specificity of the parasitic wasp *Anaphes flavipes* (Hymenoptera: Mymaridae) and a new defence in its hosts (Coleoptera: Chrysomelidae: *Oulema* spp.). Insects.

[15] Ling Z.W., Wang L.Y., Yang F.J. (2000). Study on spatial distribution pattern of rice leaf beetle (*Oulema oryzae*) in cold rice fields. Heilongjiang Bayi Agric. Univ..

[16] Császár O., Tóth F., Lajos K. (2021). Estimation of the expected maximal defoliation and yield loss caused by cereal leaf beetle (*Oulema melanopus* L.) larvae in winter wheat (*Triticum aestivum* L.). Crop Prot..

[17] Xu L.J. (1999). Study on biological characteristics and control threshold of rice leaf beetle. China Plant Prot..

[18] Zhu X.M., Zhang Q., He S.C., Gao Y., Tian Z.L. (2022). Occurrence and control of rice leaf beetle in the northeast China rice region. Agric. Technol..

[19] Kidokoro T. (1983). Migration and dispersal after hibernation in the rice leaf beetle, *Oulema oryzae* Kuwayama (Coleoptera: Chrysomelidae). Appl. Entomol. Zool..

[20] Zuo Y.L., Jin M.L. (1995). Study on the occurrence pattern of rice leaf beetle. Chin. J. Appl. Entomol..

[21] Xu C.H. (2007). Biological characteristics and control of the rice leaf beetle. North. Rice.

[22] Despland E., Hamzeh S. (2004). Ontogenetic changes in social behaviour in the forest tent caterpillar, *Malacosoma disstria*. Behav. Ecol. Sociobiol..

[23] Kher S.V., Cárcamo H.A., Evenden M.L., Dosdall L.M. (2017). Olfactory host-finding behaviour of *Oulema melanopus* (Coleoptera: Chrysomelidae) and its parasitoid, *Tetrastichus julis* (Hymenoptera: Eulophidae). J. Appl. Entomol..

[24] Truman J.W. (2019). The evolution of insect metamorphosis. Curr. Biol..

[25] Zhu Y., Wang L.Y., Yu L., Fu C.C. (2010). Effects of temperature on the growth and development of *Oulema oryzae* Kuwayama. Heilongjiang Bayi Agric. Univ..

[26] Li J., Yu G.L., Han J., Zhou B.M., Cui M.Y., Liu R.Q., Song J.W., Huang W. (2013). Study on relation between occurrence of *Oulema oryzae* and meteorological factors. Mod. Agric. Sci. Technol..

[27] Wang L.Y., Zhang H.Y., Zhu Y., Yu L., Cong B. (2011). Physiological respons of rice plants to *Oulema oryzae* feeding. Chin. J. Appl. Entomol..

[28] Wang L.Y., Cong B., Zhu Y., Yu L. (2010). Physiological responses to *Oulema oryzae* feeding cold rice variety. Hubei Agric. Sci..

[29] Wang L.Y., Jin Y.L., Zhu Y., Zhang H.Y., Ling Z.W., Qi X.H. (2016). Behavioral response of *Oulema oryzae* to secondary compounds from different rice varieties. Plant Prot..

[30] Valea I., Motegi A., Kawamura N., Kawamoto K., Miyao A., Ozawa R., Takabayashi J., Gomi K., Nemoto K., Nozawa A. (2022). The rice wound-inducible transcription factor *RERJ1* sharing same signal transduction pathway with *OsMYC2* is necessary for defense response to herbivory and bacterial blight. Plant Mol. Biol..

[31] Chung S.H., Rosa C., Scully E.D., Peiffer M., Tooker J.F., Hoover K., Luthe D.S., Felton G.W. (2013). Herbivore exploits orally secreted bacteria to suppress plant defenses. Proc. Natl. Acad. Sci. USA.

[32] Chen Y.M., Jin G.C., Liu M.Y., Wang L.L., Lou Y.G., Baldwin I., Li R. (2024). Multiomic analyses reveal key sectors of jasmonate-mediated defense responses in rice. Plant Cell.

[33] Parthiban M., Jan F.R.D., Mantoo M.A., Kaur S., Rustgi S., Wani F.J., Varshney R.K., Mir R.R. (2024). Antioxidant and secondary metabolite responses in wheat under cereal leaf beetle (*Oulema melanopus* L.) infestation. Physiol. Plant..

[34] Wielkopolan B., Szabelska-Beręsewicz A., Obrępalska-Stęplowska A. (2025). Bacteria associated with the cereal leaf beetle act as the insect’s allies in adapting to protease inhibitors, but impair its development in laboratory condition. Sci. Rep..

[35] Wielkopolan B., Krawczyk K., Szabelska-Beręsewicz A., Obrępalska-Stęplowska A. (2021). The structure of the cereal leaf beetle (*Oulema melanopus*) microbiome depends on the insect’s developmental stage, host plant, and origin. Sci. Rep..

[36] Wielkopolan B., Szabelska-Beręsewicz A., Gawor J., Obrępalska-Stęplowska A. (2024). Cereal leaf beetle-associated bacteria enhance the survival of their host upon insecticide treatments and respond differently to insecticides with different modes of action. Environ. Microbiol. Rep..

[37] Wyckhuys K.A.G., Burra D.D., Tran D.H., Graziosi I., Walter A.J., Nguyen T.G., Trong H.N., Le B.V., Le T.T.N., Fonte S.J. (2017). Soil fertility regulates invasive herbivore performance and top-down control in tropical agroecosystems of Southeast Asia. Agric. Ecosyst. Environ..

[38] Jahn G.C., Almazan L.P., Pacia J.B. (2005). Effect of nitrogen fertilizer on the intrinsic rate of increase of *Hysteroneura setariae* (Thomas) (Homoptera: Aphididae) on Rice (*Oryza sativa* L.). Environ. Entomol..

[39] Jiang M.X., Cheng J.A. (2003). Effects of fertilization levels on the white backed planthopper (Hemiptera: Delphacidae) population in rice. Chin. J. Rice Sci..

[40] Butler J., Garratt M.P.D., Leather S.R. (2012). Fertilisers and insect herbivores: A meta-analysis. Ann. Appl. Biol..

[41] Martínez L.C., Plata-Rueda A., Ramírez A., Serrão J.E. (2022). Susceptibility of *Demotispa neivai* (Coleoptera: Chrysomelidae) to *Beauveria bassiana* and *Metarhizium anisopliae* entomopathogenic fungal isolates. Pest Manag. Sci..

[42] Sun Q. (2000). Study on population dynamics and chemical control threshold of rice leaf beetle in Mishan region. Heilongjiang Bayi Agric. Univ..

[43] Kong M.F., Zuo Y.L., Liu H.X., Jin M.L., Zhu Q.Z. (1992). Study on population dynamics, damage loss, and control thresholds of rice leaf beetle in mid-season rice in the Dabie Mountains. J. Anhui Agric. Sci..

[44] Koyama J. (1978). Control Threshold for the rice leaf beetle, *Oulema oryzae* K uwayama (Coleoptera: Chrysomelidae). Appl. Entomol. Zool..

[45] Jin Y.L., Wang L.Y., Zhu Y. (2016). Sdudy of rice varieties resistant to *Oulema oryzae*. Hubei Agric. Sci..

[46] Zeng W.G., Wang L.Y., Ma Y.L. (2015). Research on the occurrence and control of rice leaf beetle and leaf miner in Qianshao farm. Mod. Agric..

[47] Konno Y., Domon K. (1998). Acetylcholinesterase sensitivity and carbamate insecticide resistance in rice leaf beetle, *Oulema oryzae* Kuwayama (Coleoptera: Chrysomelidae). Jpn. J. Appl. Entomol. Zool..

[48] Yu L., Wang L.Y., Zhang H.Y. (2011). Effects of pesticides treatment on the activity of detoxification enzymes in the larvae of *O. oryzae*. J. Anhui Agric. Sci..

[49] Shen J.K., Lu H.L. (1988). A brief report on the survey of parasitic natural enemies of rice leaf beetle in Jiangxi province. J. Environ. Entomol..

[50] Mao N., Tang P., Tian H.W., Shi M., Chen X.X. (2016). General morphology and ultrastructure of the female reproductive apparatus of *Trichomalopsis shirakii* Crawford (Hymenoptera, Pteromalidae). Microsc. Res. Tech..

[51] Ishizaki T., Ishikawa T. (2010). Pteromalid parasitoids (Hymenoptera) of *Oulema oryzae* (Kuwayama) (Coleoptera: Chrysomelidae) from Japan, with notes on *Pteromalus bifoveolatus*. Entomol. Sci..

[52] Gao J.F., Zhang L. (1994). Investigation of predators of rice leaf beetle on the southwestern slope of Changbai Mountain. Chin. J. Biol. Control.

[53] Kher S.V., Kulkarni S.S., Dosdall L.M., Cárcamo H.A. (2024). Life history and host preferences of *Tetrastichus julis* (Walker) (Hymenoptera: Eulophidae), the principal parasitoid of the cereal leaf beetle, *Oulema melanopus* (L.) (Coleoptera: Chrysomelidae). Biol. Control.

[54] Hervet V.A.D., Cárcamo H.A., Dosdall L.M., Miller T.D., Kher S.V. (2016). Assessment of potential non-target effects of *Tetrastichus julis*, a biological control agent of the cereal leaf beetle, *Oulema melanopus*. BioControl.

[55] Kheirodin A., Sharanowski B.J., Cárcamo H.A., Costamagna A.C. (2020). Consumption of cereal leaf beetle, *Oulema melanopus,* by generalist predators in wheat fields detected by molecular analysis. Entomol. Exp. Appl..

[56] Kheirodin A., Costamagna A.C., Cárcamo H.A. (2019). Laboratory and field tests of predation on the cereal leaf beetle, *Oulema melanopus* (Coleoptera: Chrysomelidae). Biocontrol Sci. Technol..

[57] Zarubova L., Kourimska L., Zouhar M., Novy P., Douda O., Skuhrovec J. (2015). Botanical pesticides and their human health safety on the example of *Citrus sinensis* essential oil and *Oulema melanopus* under laboratory conditions. Acta Agric. Scand. Sect. B-Soil Plant Sci..

