# Supplementary material for: Biological Characteristics and Research Progress of the Rice Leaf Beetle Oulema oryzae (Coleoptera: Chrysomelidae: Criocerinae)
Source: Insects. 2026 Oct 12;17(10):1050. doi: 10.3390/insects17101050 (PMC13704986; doi:10.3390/insects17101050)
Supplement: Supplementary file 1 [file insects-17-01050-s001.zip › insects-4574979-supplementary.pdf]

Supplementary materials

Table S1. Body length and width of *Oulema oryzae*.

Rice leaf beetle	Female	Male	Cocoon	4th Instar	3rd Instar	2nd Instar	1st Instar	Egg
Body length (mm)	4.42±0.26	3.97±0.25	4.83±0.39	3.82±0.32	2.07±0.20	1.45±0.17	0.87±0.12	0.71±0.05
Body width (mm)	1.58±0.06	1.43±0.07	2.30±0.17	1.48±0.08	0.94±0.21	0.7±0.06	0.37±0.03	0.34±0.02

Note: Data are presented as mean ± SD (*n* = 20 individuals per group).

Table S2. Mean ratio of the midline length of ventrite 4 to that of ventrite 5 in female and male *Oulema oryzae*.

Sex	Ratio of ventrite 4 to ventrite 5
Male	0.54 ± 0.03
Female	0.64 ± 0.04

Note: Data are presented as mean ± SD (*n* = 10 individuals per group).

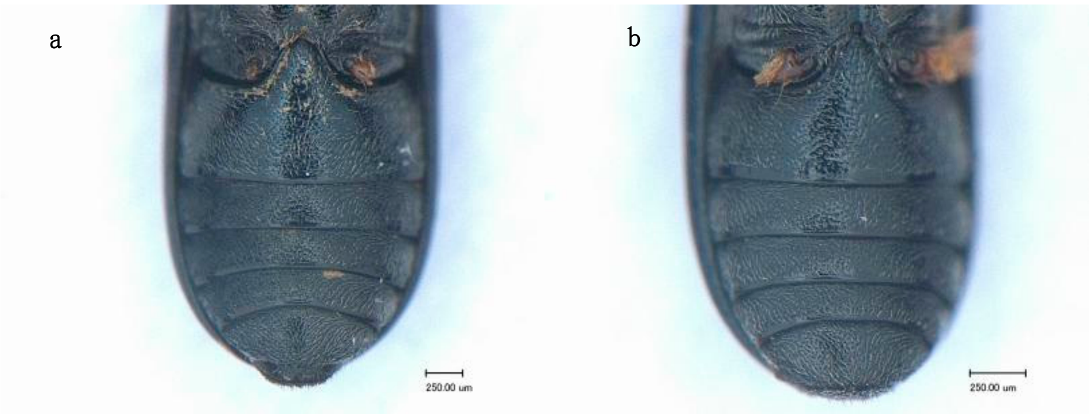

Figure S1. Abdomen of the female (**a**) and male (**b**) adult

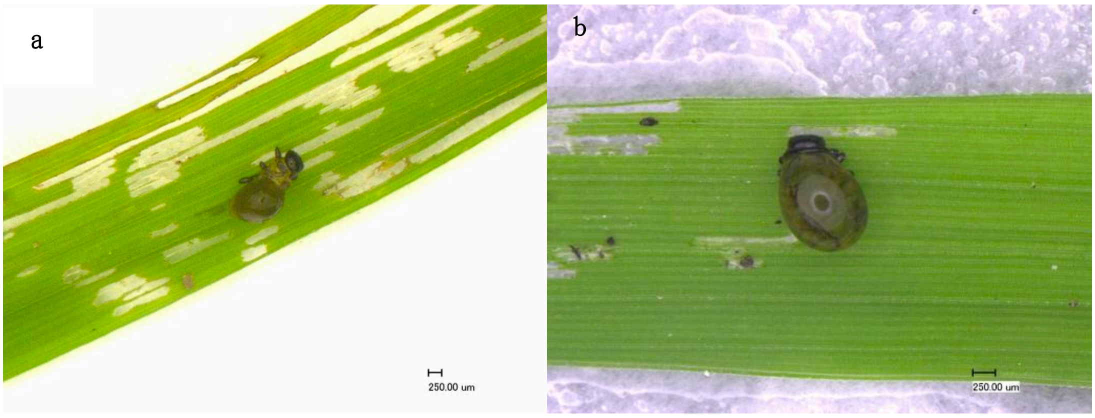

c

d

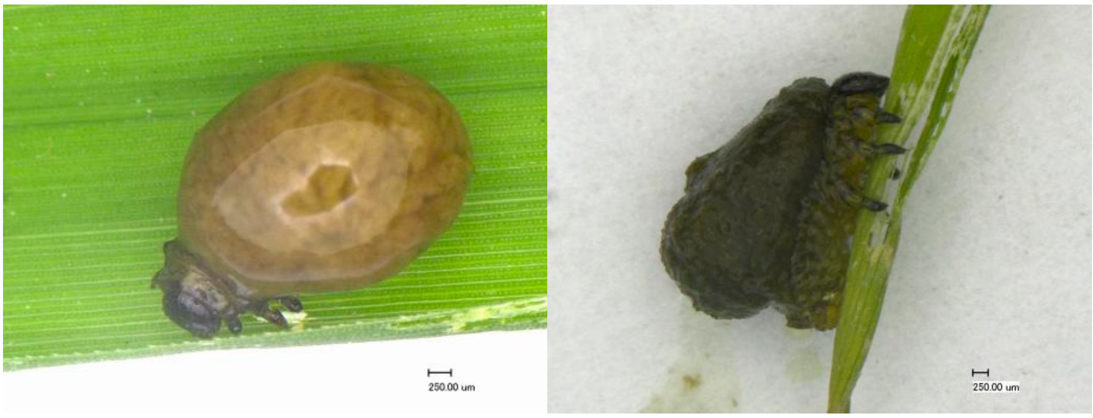

Figure S2. Mud-carrying larvae at the newly hatched (**a**), second (**b**), third (**c**), and fourth (**d**) instar stages.

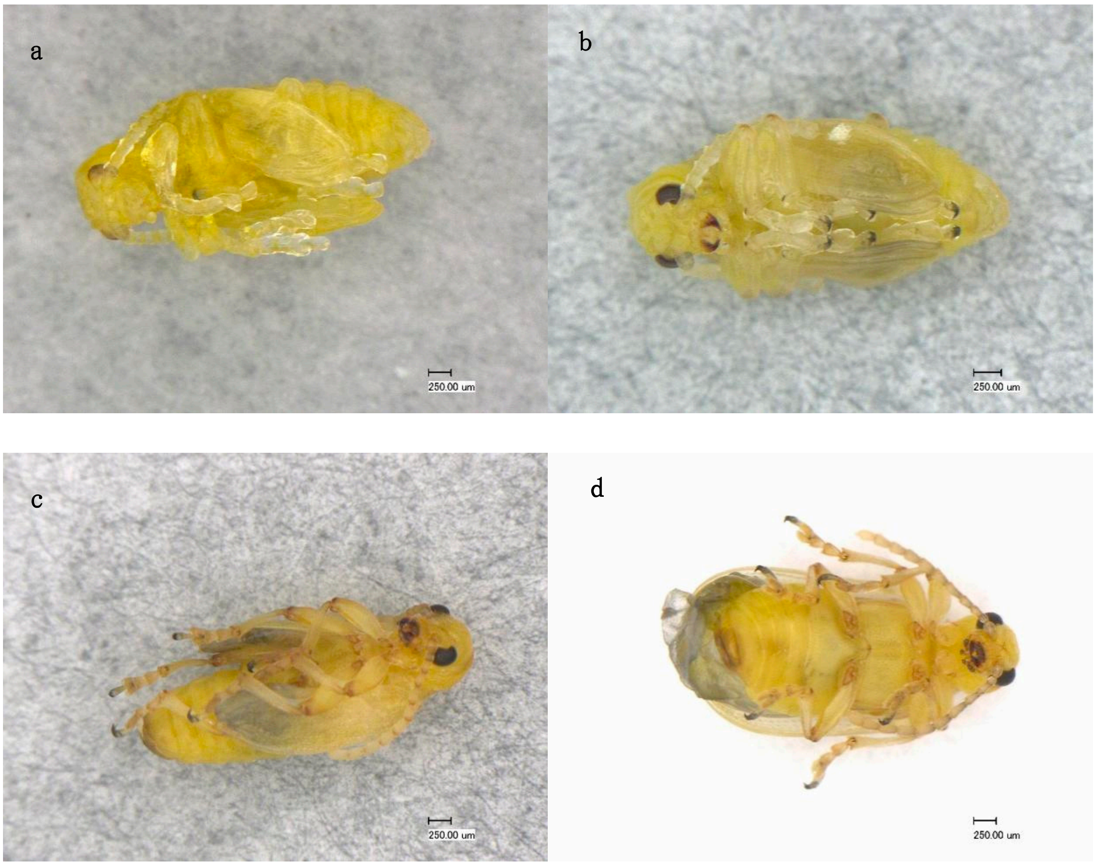

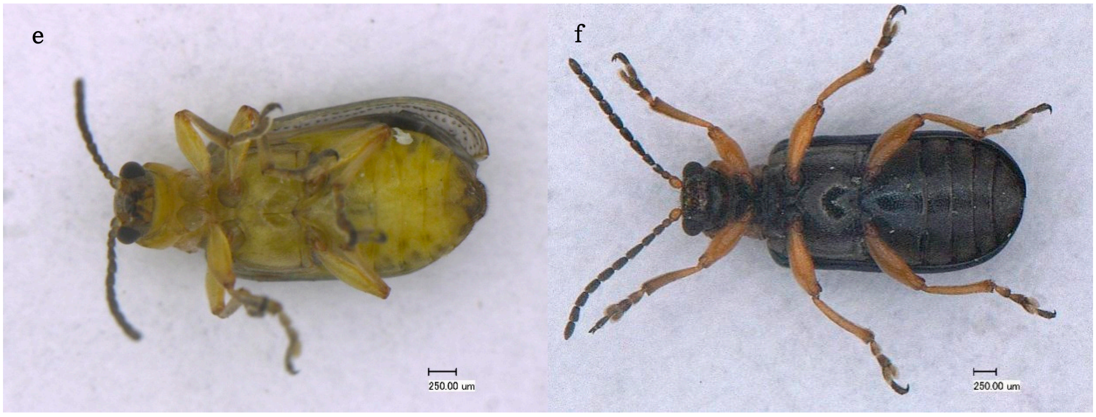

Figure S3. Abdomen of 4–8-day post-eclosion adults(**a-e**) and newly emerged adults(**f**) of the rice leaf beetle (*Oulema oryzae*).
